# Is there a Role for Anti-IL-5 Therapies in Eosinophilic Fasciitis?

**DOI:** 10.31138/mjr.301223.itr

**Published:** 2023-12-30

**Authors:** Andreas Angelopoulos, Ioannis Kouverianos, Dimitrios Daoussis

**Affiliations:** 1 Internal Medicine Resident, General Hospital of East Achaia, Kalavryta, Greece,; 2 Medical Graduate, University of Patras Medical School, Patras, Greece,; 3 Department of Rheumatology, Patras University Hospital, University of Patras Medical School, Patras, Greece

**Keywords:** eosinophilic fasciitis, EF, groove sign, mepolizumab, benralizumab, reslizumab

## Abstract

**Introduction:**

Eosinophilic Fasciitis (EF) is a rare disease, originally proposed as “diffuse fasciitis with eosinophilia” by Shulman in 1974. Symptoms of EF include peripheral eosinophilia accompanied by symmetrical inflammation of the subcutaneous fascia and muscle, usually locating in the upper arms or thighs. There is no approved standard of care treatment.

**Methods:**

Taking into account that eosinophils may be pathogenetically involved in EF, we performed a review on Medline focusing on anti-Interleukin-5 (IL-5) therapies in EF.

**Results:**

Only one case of a patient with EF has been reported who was successfully treated with reslizumab, an anti-IL-5 therapy. The patient had EF refractory to the commonly used immunosuppressive treatment but when reslizumab was added, the patient experienced remission of her symptoms.

**Discussion:**

The exact aetiology of EF is still unclear, and many therapeutic approaches have been tested. Commonly used immunosuppressive agents, such as corticosteroids are not always effective and associate with significant side effects. Eosinophils seem to have a role in the pathogenesis of the disease; anti-eosinophilic therapies targeting IL-5/IL-5 Receptor could be an attractive alternative for the treatment of the disease.

## INTRODUCTION

Eosinophilic Fasciitis (EF) is a rare autoimmune disease of unknown aetiology and unclear pathogenesis. Hitherto, less than 300 cases have been reported since 1974, when Shulman first discovered the disease.^1,2^ EF has an acute onset and it is characterised, in its early stage, by symmetrical inflammation of the subcutaneous fascia of the limbs or trunks and later by thickening of the collagen of the fascia with a characteristic clinical phenotype. Peripheral eosinophilia is found in the majority of cases and eosinophilic infiltration of the subcutaneous fascia is a hallmark of the disease. The treatment of EF includes mainly oral corticosteroids, and in some cases, other immunosuppressive agents such as methotrexate. Taking into account that eosinophils may play a crucial role in the pathogenesis of the disease in its early stage, alternative and corticosteroid-sparing therapies that could deplete eosinophils should be explored for the therapy of EF. Interleukin 5 (IL-5) is the major cytokine for the maturation, differentiation, proliferation, and migration of eosinophils; thus, it is worthwhile to examine whether monoclonal antibodies against IL-5 or IL-5 Receptor could potentially have a supplementary role in the treatment of EF.^2–5^ Of note, anti-eosinophilic therapies are already used in a wide range of diseases with an acceptable safety profile.^6^

## OVERVIEW OF THE DISEASE

Although only 300 EF cases have been reported so far, the real number of EF patients could be much higher due to misdiagnosis and underreporting. EF usually appears in ages between 20-60 years, but it may also appear in children.^4^ The average time to diagnose EF is approximately 11 months.^7^

The aetiology of EF is still not elucidated but it has been reported that a significant proportion of patients have a specific background before the onset of EF. Many patients (28–46%) report a history of strenuous exercise or trauma before the development of symptoms.^2,3,7–9^ Other less common aetiological factors are infection with Borrelia burgdorferi, radiation therapy, treatment with specific agents such as Nivolumab, autoimmune diseases such as Hashimoto’s thyroiditis and diabetes mellitus, and haematologic disorders.^10–16^

**Figure 1.**
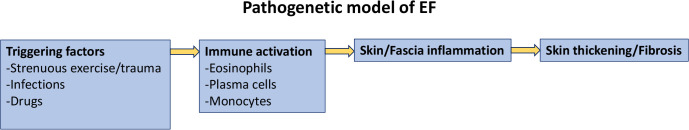
Proposed pathogenetic mechanism.

EF usually starts with abrupt symmetrical swelling and erythema of arms/legs with involvement of the skin and the deeper peri muscular fascia accompanied by eosinophilia in peripheral blood. The early stage of the disease is characterized by pain and systemic symptoms such as fever and fatigue. The lesions are located in the upper arms and thighs and never in the face or fingers.^4^) The latter are affected only in Systemic sclerosis (SSc) and not in EF or localised scleroderma, which is a clinical differentiation sign. Raynaud’s Phenomenon, a typical manifestation of SSc is characteristically absent in EF. As the disease progresses, the redness and oedema of the skin are gradually transformed to a ‘peau d’ orange’ consistency and the general symptoms include weight loss and myalgia. Furthermore, due to the thickening of the peri muscular fascia, the range of motion of nearby joints may be reduced. The infiltration of fascia with leukocytes and the development of fibrosis causes the skin to become harder and the soft blood vessels to pull inwards creating the “groove sign”. Groove sign, which is a hallmark of the disease, is visible when the patient elevates his/her limb above the level of the heart, due to increased blood return.^4,8,17–18^ Another clinical manifestation of EF, when the upper limbs are affected, is peripheral neuropathy, usually carpal tunnel syndrome.^9,19^ The proposed pathogenetic model is depicted in **Figure 1**.

Laboratory findings include eosinophilia and high IL-5 levels in the blood stream, especially in the early stage of the disease, hypergammaglobulinemia, elevated C-Reactive Protein (CRP) and Erythrocyte Sedimentation Rate (ESR).^2,4,15,20^ Increased serum aldolase may be a useful indicator of disease activity as it seems that aldolase levels decrease to the normal range after oral corticosteroid treatment and tend to increase again after the recurrence of skin sclerosis.^21,22^ A significant number of EF cases are associated with haematological abnormalities (10%) and solid organ tumours such as breast, lung, prostate cancer and melanoma.^15,23^ Associated haematological disorders include severe aplastic anaemia, thrombocytopenia, myeloproliferative disorders, and lymphocytic leukaemia.^9,14^ The gold standard for the diagnosis of EF is a full-thickness biopsy which includes the muscle and the fascia. Histology reveals inflammatory infiltration of the fascia with eosinophils, plasma cells and macrophages, whereas myositis is often observed. As the disease progresses the biopsy findings show thickening of the fascia and the underlying tissue as well as development of fibrosis. Magnetic Resonance Imaging (MRI) has been recently used to determine the extent of inflammation and evaluate the response to treatment.^2,3,8^

The cornerstone therapy of EF is oral corticosteroids which are effective in the 90% of the reported cases, with a starting dose of 20-30 mg/day. Some patients have steroid-refractory EF and alternative therapies have been used. These therapies include methotrexate, cyclosporin, cyclophosphamide and recently rituximab.^24–27^ Psoralen and ultraviolet A (PUVA) therapy have also shown efficacy in EF.^28^

## METHODS

We performed an electronic search on Medline and Scopus using the following keywords: eosinophilic fasciitis, EF, groove sign, mepolizumab, benralizumab, and reslizumab. Our search included papers published from 1974 and onwards. Our main focus was to investigate whether patients with EF have received anti-IL-5 therapies and if this treatment was effective and safe. Our research was carried out from September 2022 to February 2022 and focused on papers published in the last 5 years. Because of the rarity of the disease, we mostly found case reports and articles about clinical manifestations of the disease.

## RESULTS

Our search identified only a single case where anti-eosinophilic therapy has been used in EF. The patient was 65 years old with a history of strenuous exercise and was diagnosed with EF based on clinical manifestations, peripheral eosinophilia, and skin biopsy which included the fascia and inflamed muscle. The initial therapy was 60 mg prednisone combined with hydroxychloroquine 200 mg twice a day as a steroid-sparing agent. Methotrexate in a dose of 25 mg weekly and mycophenolate 1000 mg twice a day were added when steroids were tapered below 10 mg but the patient experienced ongoing symptoms of skin tightness. Thus, the authors explored whether an anti-IL-5 agent could possibly promote disease remission. The Food and Drug Administration (FDA) has approved three anti-IL-5 agents; mepolizumab, benralizumab, and reslizumab. In our case, the latter was chosen due to its weight-based dosing. Reslizumab was delivered at a dose of 3 mg/kg intravenously every 4 weeks and led to improvement of her symptoms. This therapy led to the discontinuation of prednisone for the last 2 years, tapering of methotrexate dose to 15 mg weekly, remission of the activity of the disease and to a better quality of patient’s life.^5^

## DISCUSSION

Eosinophilic fasciitis is characterised by peripheral eosinophilia and eosinophilic infiltration of the muscle and the fascia, even though eosinophils are not always present in the histopathology.^2^ Thus, there is no direct proof for the pathogenetic role of eosinophils in EF. Eosinophils are present in the early stage of the disease which is confirmed by full-thickness biopsy of the inflamed tissue. Therefore, eosinophils and/or IL-5 or IL-5 Receptor could be an alternative therapeutic target. The existing therapies are not always effective and associate with adverse events. Refractory disease occurs in a proportion of patients and there are no current guidelines or approved therapies for this condition. Targeting IL-5 which promotes the growth, maturation, and migration of eosinophils is capable of reducing the total number of eosinophils in the blood stream and in affected tissue. The successful outcome of a patient with EF, treated with reslizumab, paved the way for new trials with anti-IL-5 therapies. An upcoming exploratory study is going to investigate the efficacy and safety of mepolizumab, an anti-IL-5 agent, in the treatment of EF. This study is going to recruit 6 EF patients and treat them with 400 mg of mepolizumab administrated subcutaneously every 4 weeks for 24 weeks (NCT04305678). Up until then anti-eosinophilic therapies may be considered in highly selected cases refractory to conventional treatments. Further research needs to be conducted regarding the potential pathogenetic role of eosinophils in the disease.
